# Swelling Mechanism of Rubber Sealing Materials in Methanol Transportation Pipelines

**DOI:** 10.3390/ma19101984

**Published:** 2026-05-11

**Authors:** Zitao Jiang, Zigeng Huang, Gengsheng Chen, Yunan Zhang, Shimao Liu, Ziru Chang, Xinru Yang

**Affiliations:** 1 College of Mechanical and Transportation Engineering, China University of Petroleum (Beijing), No. 18 Fuxue Road, Changping District, Beijing 102249, China; jiangzt@cup.edu.cn (Z.J.); chengs@blueskyoil.com (G.C.); 2024210801@student.cup.edu.cn (S.L.); 2023210793@student.cup.edu.cn (Z.C.); 2023215593@student.cup.edu.cn (X.Y.); 2 National Center for Materials Service Safety, University of Science and Technology Beijing, No. 30 Xueyuan Road, Haidian District, Beijing 100083, China; zhangyn@ustb.edu.cn

**Keywords:** methanol pipeline, sealing materials, swelling behavior, plasticizer migration, dissolution mechanism

## Abstract

The growing demand for long-distance green methanol transportation highlights the critical need to evaluate the safety and reliability of pipeline sealing materials. This study investigates the swelling mechanisms of fluorocarbon rubber (FKM), nitrile butadiene rubber (NBR), and polytetrafluoroethylene (PTFE) under simulated methanol pipeline conditions. Static immersion tests were conducted under simulated pipeline conditions with water contents of 0–20% and temperatures of 25–55 °C, supplemented by thermogravimetric analysis (TGA), Fourier transform infrared spectroscopy (FTIR), and gas chromatography–mass spectrometry (GC–MS). FKM exhibited severe physical swelling, with the volume increase reaching up to 80% in pure methanol. Notably, the addition of 5% water markedly suppressed this swelling, reducing the volume change of FKM sealing rings to approximately 3% and the mass change to 1%. Conversely, NBR experienced volume shrinkage and mass loss due to the extraction of the plasticizer Bis(2-ethylhexyl) phthalate by methanol, a process also inhibited by water. PTFE demonstrated exceptional chemical stability and negligible dimensional changes owing to its high crystallinity and rigid structure. Consequently, PTFE is recommended as the optimal sealing material for pure methanol pipelines. When utilizing FKM or NBR, strict control over the fluid’s water content and operating temperature is essential to prevent degradation and ensure long-term pipeline integrity.

## 1. Introduction

As a critical basic chemical raw material and clean energy source, methanol holds significant strategic importance. Against the backdrop of global carbon neutrality, the methanol industry is facing new opportunities for green transformation. Green methanol, produced by synthesizing hydrogen from renewable energy with carbon dioxide or through biomass gasification, possesses significant low-carbon advantages [1,2,3]. Furthermore, acting as an excellent liquid carrier for hydrogen energy, green methanol effectively resolves the hydrogen embrittlement issues often encountered during hydrogen storage and transportation, thereby promoting the optimization of energy structures and sustainable development.

In recent years, green methanol has shown broad application prospects in fields such as transportation fuels, marine propulsion, and energy storage, attracting widespread attention for its high octane number and low pollution emission characteristics [4,5]. The international shipping industry has identified green methanol as a key option for emission reduction, and several countries have already initiated the operation of demonstration fleets [6]. Driven by both policy incentives and technological breakthroughs, China has initially formed an industrial ecosystem for green methanol, with new energy-coupled methanol projects laid out in multiple regions, laying the foundation for large-scale industrial development [7].

However, there is a highly unbalanced geographical distribution between methanol production centers and consumption markets. Characterized by resource endowments of “rich coal, poor oil, and little gas,” China’s methanol industry relies primarily on coal-based production. Production capacity is concentrated in the resource-rich Northwest and Northeast regions—such as Inner Mongolia, Ningxia, Xinjiang, Shaanxi, and Jilin—where the construction of new energy-coupled methanol projects is being vigorously promoted by leveraging abundant coal, wind, and solar resources [1,6]. In contrast, methanol consumption markets are mainly concentrated in the economically developed eastern and southern coastal regions, such as Shandong, Jiangsu, Zhejiang, and Guangdong [4,7]. This geographical mismatch between production and consumption has led to a significant increase in demand for long-distance methanol transportation.

Current transportation methods mainly rely on road, rail, and water transport, which suffer from high costs, low efficiency, and poor safety and environmental performance. In comparison, long-distance methanol pipelines offer significant advantages, including high safety, strong continuity, and low transport costs, enabling efficient resource circulation while reducing carbon emissions and transportation risks.

Nevertheless, the presence of polar hydroxyl groups in methanol molecules imparts strong swelling properties on rubber sealing materials, easily inducing changes in material mass and volume as well as a decline in mechanical properties. Existing studies have largely focused on methanol-gasoline blends, with a lack of research on the swelling mechanisms of pure methanol under pipeline transportation conditions [8,9,10].

Utilizing existing long-distance pipelines for methanol transportation places high demands on sealing materials currently in service. In long-distance pipeline systems, swelling of sealing materials may become more pronounced under the combined effects of pressure, temperature variation, and long-term methanol immersion, thereby increasing the risk of leakage and safety accidents. Accordingly, the objective of this work is to evaluate the applicability and degradation mechanisms of representative sealing materials for long-distance methanol pipeline service. The scope of this study covers three commonly used sealing materials, namely fluorocarbon rubber (FKM-26), nitrile butadiene rubber (NBR-7006), and polytetrafluoroethylene (PTFE-JF-G90), exposed to pure methanol and methanol–water mixtures with water contents of 0–20%, and to temperatures of 25–55 °C under simulated pipeline conditions. The work compares the mass and volume changes of these materials, identifies material-specific degradation characteristics using TGA, FTIR, and GC–MS analyses, and further discusses the swelling mechanism of FKM based on Hansen solubility parameter analysis. Furthermore, reliable experimental data are also necessary for future data-driven prediction and advanced material design, including machine-learning-assisted swelling analysis and the development of self-healing or nanocomposite elastomers for improved sealing durability [11,12].

## 2. Materials and Methods

### 2.1. Materials and Instruments

Methanol (purity 99.99%) was purchased from Shanghai Titan Scientific Co., Ltd. (Shanghai, China).

Rubber O-rings selected for this study included Nitrile Butadiene Rubber (NBR-7006), Fluorocarbon Rubber (FKM-26), and Polytetrafluoroethylene (PTFE-JF-G90). Additionally, spare NBR and FKM sealing rings from the Ruhr pumps of the Lan-Zheng long-distance oil pipeline were used as field samples for comparative analysis.

The instruments used in this study include:

- Thermogravimetric Analyzer (TGA): Discovery TGA 550, TA Instruments (New Castle, DE, USA), with data processed using Trios software (Version 5.1.1.46572)
- Fourier Transform Infrared Spectrometer (FTIR): TENSOR II, Bruker Scientific Instruments (Hong Kong) Co., Ltd. (Hong Kong, China), equipped with OPUS software (Version 7.5) for spectral analysis;
- Electronic Balance: BSA224S, Sartorius Scientific Instruments (Beijing) Co., Ltd. (Beijing, China);
- Gas Chromatography-Mass Spectrometry (GC-MS): Agilent 8860, analyzed via Agilent MassHunter GC/MS Acquisition software (Version 10.2.489).

Additionally, all experimental data visualization, peak fitting, and statistical analyses were performed using OriginPro 2021 software (OriginLab Corporation, Northampton, MA, USA).

**Figure 1:**
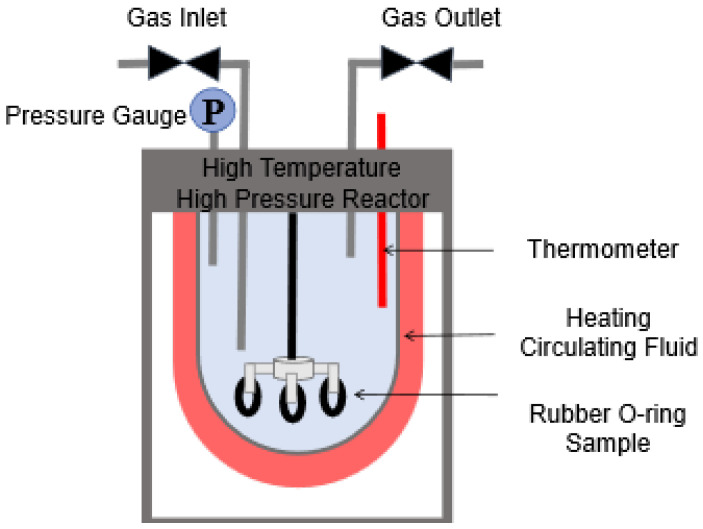
Schematic diagram of the high-temperature and high-pressure reactor.

Figure 1 shows the high-temperature high-pressure reactor used for static immersion tests, which had an effective volume of 3 L.

### 2.2. Experimental Methods

The static immersion method was employed for the experiments. Prior to immersion, the rubber O-rings were weighed using an electronic balance with a precision of 0.1 mg, and the mass in air ($m_{i}$) and the mass in distilled water ($m_{i,w}$) were recorded. The samples were completely immersed in methanol or methanol–water solutions in a sealed high-temperature and high-pressure reactor with an effective volume of 3 L. For each test, 2 L of methanol or methanol–water solution was added to the reactor to ensure that the specimens were fully immersed throughout the immersion process. During the immersion tests, the reactor was sealed to minimize methanol evaporation and maintain the preset pressure. The immersion pressure was maintained at 4 MPa. The samples were immersed for 7 days. To simulate the conditions of long-distance pipeline transportation, the experimental parameters were set as follows: water content ranging from 0% to 20%, and temperature ranging from 25 °C to 55 °C. Three samples were selected for each of the three sealing materials: Nitrile Butadiene Rubber (NBR), Fluorocarbon Rubber (FKM), and Polytetrafluoroethylene (PTFE). For each condition, three parallel specimens were tested, and the results are reported as mean values ± standard deviation. While a sample size of n=3 is acceptable for establishing consistent trends in such static immersion tests, the authors acknowledge that a limited sample size restricts the broader statistical power, which stands as a limitation of the current quantitative evaluation.

After immersion, the mass of each sample in air ($m_{0}$) and in distilled water ($m_{0,w}$) was measured using the electronic balance, and the data were recorded. The volume change in the rubber was calculated according to Equation (1). The volume change was calculated based on the Archimedes buoyancy principle.

(1) $$\Delta V=\frac{\left(m_{0}-m_{0,w}\right)-\left(m_{i}-m_{i,w}\right)}{m_{i}-m_{i,w}}\times 100\%$$

where

ΔV—the volume change ratio;
$m_{0}$—Mass of the sample in air after immersion (g);
$m_{0,w}$—Mass of the sample in distilled water after immersion (g);
$m_{i}$—Mass of the sample in air before immersion (g);
$m_{i,w}$—Mass of the sample in distilled water before immersion (g).

Typical non-metallic materials from oil and gas pipelines were processed into rubber sheets for grouping, and their original dimensions and shapes were recorded. These samples were then immersed in a reactor filled with 2 L of methanol for 24 h. Upon completion of the experiment, the rubber sheets were retrieved to observe changes in shape, volume, and color. The magnitude of swelling was evaluated by the ratio of the dimensional change before and after the test to the original dimensions. The 24 h immersion test was used as a short-term visual screening test to evaluate the initial dimensional and appearance changes of typical non-metallic materials, whereas the 7 d immersion test was used for quantitative evaluation of the mass and volume changes of sealing rings under simulated pipeline conditions.

### 2.3. Thermogravimetric Analysis (TGA)

Thermogravimetric analysis (TGA) was employed to evaluate the overall structural changes of the sealing rings before and after immersion. The O-rings of the three materials, after being immersed in methanol solutions with varying water contents, were cut into small pieces for TGA testing under a pure nitrogen (N_2_) atmosphere. The sample mass ranged from approximately 5 to 30 mg. The specific test parameters were as follows: a nitrogen flow rate of 50 mL/min, a protective gas (pure N_2_) flow rate of 20 mL/min, and a temperature range from 40 °C to 750 °C with a heating rate of 10 °C/min [13].

### 2.4. Infrared Spectroscopy Measurement (FTIR)

Infrared absorption spectroscopy was utilized to analyze the changes in functional groups of the three rubber materials following immersion in methanol. Specifically, the measurements were conducted in Attenuated Total Reflection (ATR-FTIR) mode, with a wavenumber range from 4000 cm^−1^ to 650 cm^−1^ and a resolution of 4 cm^−1^.

### 2.5. Gas Chromatography–Mass Spectrometry (GC-MS) Analysis

Gas Chromatography–Mass Spectrometry (GC-MS) was employed to analyze the compositional changes of the methanol solutions following the immersion of the rubber materials. The immersion fluids obtained from the three rubber materials, which had been soaked in pure methanol at atmospheric pressure and 50 °C for 7 days.

For GC–MS analysis, the methanol solution collected after rubber immersion was used as the test sample, rather than the rubber specimen itself. Before GC–MS measurement, the dissolved leachates in the methanol solution were enriched to improve the detectability of trace compounds. Compound identification was performed by comparing the obtained mass spectra with the NIST mass spectral library, together with retention-time information. Only peaks with reliable library matching were considered in the qualitative analysis.

The chromatographic conditions were as follows: A DB-5MS capillary column (30 m × 0.25 mm × 0.25 µm) was utilized. The inlet temperature was set to 280 °C for the FKM and PTFE samples, and 250 °C for the NBR samples. The oven temperature program was initiated at 50 °C (held for 2 min), then ramped at a rate of 10 °C/min to 300 °C, where it was held for 5 min. Helium was used as the carrier gas at a constant flow rate of 1.2 mL/min. The injection mode was split with a ratio of 1:10. The mass spectrometry conditions included an Electron Impact (EI) ionization source at 70 eV, with a mass scan range of *m*/*z* 35–500.

## 3. Results and Discussion

### 3.1. Mass and Volume Changes in Rubber Under Different Water Contents in Methanol

**Figure 2:**
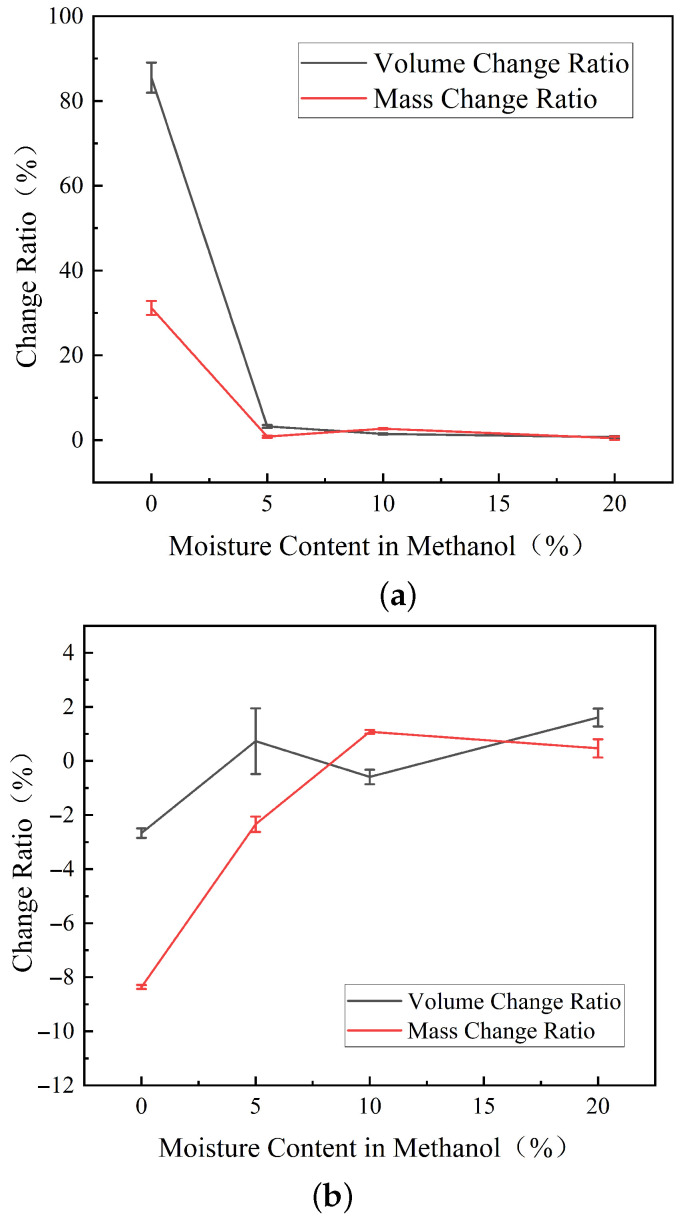

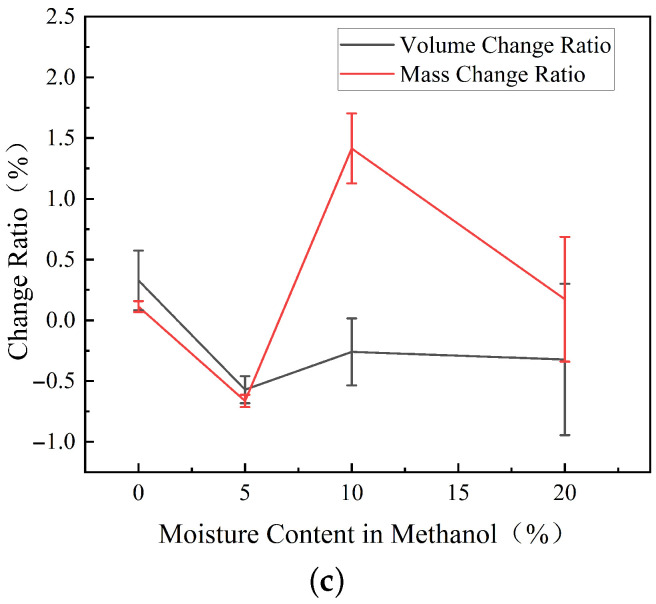
Mass and volume changes in rubber samples under different water contents in methanol: (**a**) FKM; (**b**) NBR; (**c**) PTFE.

The mass and volume changes in the rubber samples under varying moisture contents in methanol are presented in Figure 2. For FKM, the average volume change decreased from 85.49±3.56% in pure methanol to 3.25±0.32% at 5% water content, while the average mass change decreased from 31.19±1.65% to 0.82±0.27%. This indicates that 5% water content markedly reduced both volume expansion and mass uptake of FKM. For NBR, the mass change increased from −8.43±0.14% in pure methanol to nearly zero at 10% water content. With increasing water content, the influence on volume and mass changes in NBR was progressively reduced. For PTFE, both mass and volume changes remained within a narrow range under different water contents.

Specifically, when immersed in pure methanol, FKM exhibits a maximum volume change rate of up to 80%, which far exceeds the maximum acceptable range for rubber volume change (+25%/−5%) stipulated by the standard GB/T 34903.2-2017 [14]. Molecular chains in vulcanized rubber typically aggregate loosely, creating free volume and interstitial spaces within the molecular network. Solvents can penetrate these gaps, leading to volume expansion and mass increase, a phenomenon known as swelling [15,16].

The affinity of rubber for liquid absorption is determined by its monomer composition, and the polarity of the rubber is critical for its compatibility with the liquid [17,18,19]. This compatibility is often expressed by the solubility parameter (δ), a thermodynamic property related to cohesive energy density. Substances with identical or similar δ values are likely to exhibit high affinity for one another. Therefore, if the δ value of a liquid matches or is close to that of the rubber, the likelihood of absorption increases [20].

To quantitatively evaluate the interaction between methanol and FKM-26, the Hansen solubility parameter (HSP) theory was adopted. The Hansen solubility parameters for the materials studied are listed in Table 1. HSP divides the cohesive energy into three components: dispersion ($\delta_{D}$), polar ($\delta_{P}$), and hydrogen-bonding ($\delta_{H}$). The total solubility parameter is expressed as:

(2) $$\delta_{t}={\left(\delta_{D}^{2}+\delta_{P}^{2}+\delta_{H}^{2}\right)}^{1/2}$$

The affinity between a solvent and a polymer can be described by the Hansen distance $R_{a}$:

(3) $$R_{a}={\left[4{\left(\delta_{D,s}-\delta_{D,p}\right)}^{2}+{\left(\delta_{P,s}-\delta_{P,p}\right)}^{2}+{\left(\delta_{H,s}-\delta_{H,p}\right)}^{2}\right]}^{1/2}$$

The relative energy difference (RED) is defined as:

(4) $$RED=\frac{R_{a}}{R_{o}}$$

where $R_{o}$ represents the interaction radius of the polymer. In general, RED<1 indicates good compatibility, while RED>1 suggests poor thermodynamic affinity [21].

The HSP values of the mixed solvent were estimated using a linear volume-fraction approximation:

(5) $$\delta_{D,\mathrm{mix}}=15.1+0.4x$$

(6) $$\delta_{P,\mathrm{mix}}=12.3+3.7x$$

(7) $$\delta_{H,\mathrm{mix}}=22.3+20x$$

where *x* is the volume fraction of water. This approach assumes that the cohesive energy density of the mixture can be expressed as the weighted sum of its components and is widely used in Hansen solubility theory for multicomponent systems [21]. However, it should be noted that this method represents an approximation and may deviate from the actual behavior for strongly interacting systems such as methanol–water mixtures. Specifically, the strong intermolecular hydrogen bonding between methanol and water can lead to non-ideal mixing, forming dynamic molecular complexes and resulting in excess molar volumes. Consequently, the actual cohesive energy density of the binary mixture may not perfectly align with the linear volume-fraction weighted sum, which introduces a certain degree of theoretical deviation when predicting the Hansen distance.

Compared to hydrogen atoms, fluorine atoms in FKM possess a larger van der Waals radius (∼1.47 Å) and high electronegativity (3.98), resulting in bulky side groups that restrict chain rotation and segmental mobility, thereby enhancing molecular rigidity and chemical stability. Despite this rigidity, methanol is still able to penetrate into the FKM network due to its small molecular size and high diffusivity.

From the HSP analysis, the Hansen distance between methanol and FKM-26 is $R_{a}=20.85$ MPa^1/2^, corresponding to RED=2.37, indicating poor thermodynamic compatibility. With increasing water content, both $R_{a}$ and RED increase further, suggesting that the addition of water reduces the overall affinity between the solvent system and FKM. The calculated values for the different solvent systems are summarized in Table 2.

Furthermore, the interaction radius $R_{o}$ of FKM-26 was approximated as 8.8 MPa^1/2^ based on literature values obtained from experimental swelling-based Hansen sphere fitting of fluorocarbon elastomers [22]. It should be noted that $R_{o}$ is an empirical parameter rather than a universal constant; it exhibits a characteristic “zone of uncertainty” (8.0–12.0 MPa^1/2^) and is highly sensitive to polymer composition, filler content, and crosslink density, which restrict macromolecular mobility [23].

Although the calculated RED values suggest poor compatibility, small molecular solvents such as methanol may behave as apparent “outliers”, exhibiting stronger interaction with polymers than predicted by solubility parameter distance alone due to their low molar volume and enhanced diffusion capability [21]. Consequently, methanol can still diffuse into the free volume of FKM.

Once absorbed, methanol acts as a plasticizer, disrupting weak intermolecular interactions (such as van der Waals forces), increasing chain mobility, and reducing the glass transition temperature ($T_{g}$). This plasticization effect enhances solvent diffusion and accelerates swelling. In contrast, water, which possesses a much higher hydrogen-bonding parameter, exhibits poor affinity with FKM and primarily acts as a diluent in the mixed solvent system, thereby reducing methanol uptake and suppressing swelling.

**Figure 3:**
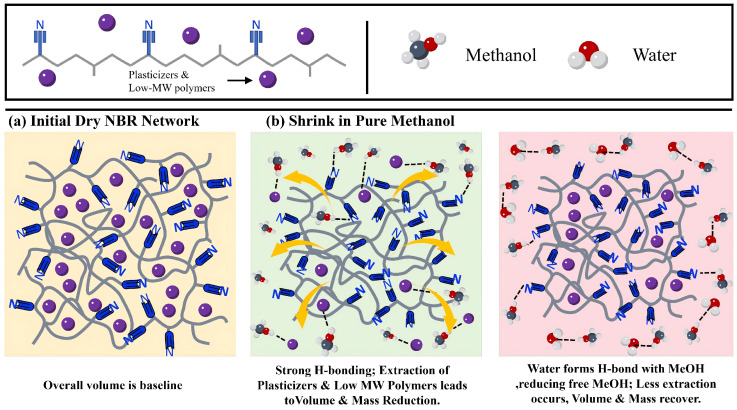
Schematic diagram of the swelling mechanism of nitrile butadiene rubber (NBR). The arrows represent the extraction of substances (such as plasticizers and low-molecular-weight polymers) from the rubber matrix into the solution.

Conversely, the volume and mass changes in NBR in pure methanol are opposite to those of FKM. After 7 days of immersion in pure methanol, both the volume and mass of NBR decreased. As the water content increased, the rates of change for NBR’s volume and mass gradually rose, returning to near-initial levels. This phenomenon occurs because methanol is highly polar, while the nitrogen elements in the polar groups of NBR possess lone pair electrons. These electrons create electrostatic interactions with the hydrogen in methanol, facilitating the formation of N-H hydrogen bonds and the adsorption of methanol molecules. When the methanol concentration is sufficiently high, the strength of the hydrogen bonds between the polar groups of NBR and methanol increases, causing low-molecular-weight polymers within the NBR network to dissolve into the methanol solution [24]. Similarly, substances such as plasticizers in the NBR are extracted into the solution [25], resulting in a reduction in the overall volume and mass of the NBR. As the water content increases, water molecules readily form hydrogen bonds with methanol, reducing the concentration of free methanol molecules and lowering the overall polarity of the solution. Consequently, the extraction effect diminishes, and the mass and volume of the NBR recover. The swelling mechanism of NBR is shown in Figure 3.

The variation in water content had little influence on the mass and volume changes in PTFE, indicating that PTFE remained dimensionally stable in both pure methanol and methanol–water mixtures. This stability is mainly associated with its highly fluorinated linear chain structure, strong C–F bonds, high crystallinity, compact chain packing, and limited free volume. These structural features hinder the diffusion of methanol molecules into the polymer matrix and suppress solvent-induced plasticization or chain expansion. Consequently, neither pure methanol nor methanol–water mixtures caused obvious macroscopic swelling of PTFE. Compared with FKM and NBR, PTFE therefore exhibited the highest resistance to methanol-induced dimensional changes.

**Figure 4:**
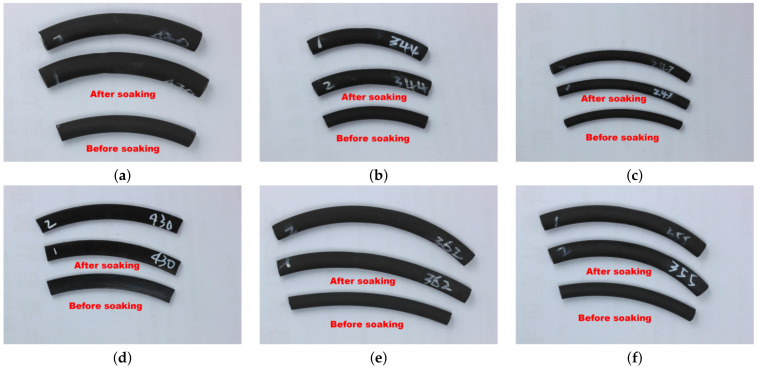
Comparison of rubber field samples before and after immersion: (**a**) FKM 430; (**b**) FKM 344; (**c**) FKM 247; (**d**) NBR 430; (**e**) FKM 362; (**f**) FKM 355.

Based on the experimental finding that FKM and NBR are more susceptible to swelling, six types of actual sealing rings used in Ruhr pumps for pipeline transmission were collected for simulated testing. The conditions were controlled at 0% water content and 25 °C. The morphology of the immersed sealing rings is shown in Figure 4. It was observed that all FKM samples exhibited varying degrees of swelling, with FKM (430) showing the most significant change. The NBR (430) showed almost no change after immersion. These field results confirm that FKM materials are more prone to swelling than NBR materials in practical applications.

**Figure 5:**
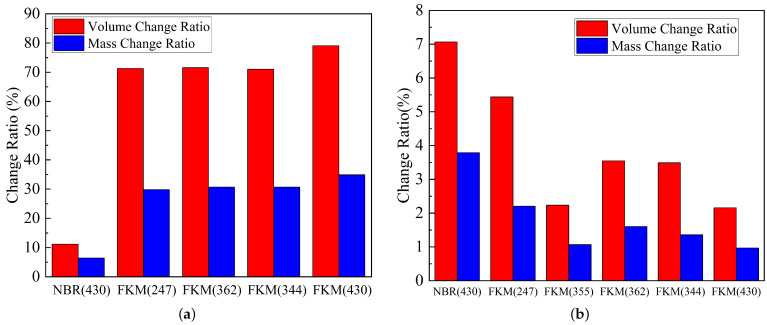
Swelling comparison of field sealing rings: (**a**) Pure methanol solution; (**b**) 5% Water content.

Simulation experiments were further conducted by controlling the water content in the methanol solution at 5% and comparing the results with the 0% water content data, as shown in Figure 5. In pure methanol solution, FKM (430) exhibited the most pronounced swelling, with a volume change rate of +79% and a mass change rate exceeding +30%. Other FKM samples showed volume change rates of approximately +70% and mass change rates of around +30%, consistent with morphological observations. The field NBR (430) sample showed slight swelling, with a volume change rate of approximately +12% and a mass change rate of about +6%. This differs from the results of the commercial NBR rubber tested earlier (which shrank), a discrepancy likely attributable to differences in rubber formulation and material grade.

With the addition of 5% water, the swelling of all sealing rings was inhibited. The inhibition was most severe for FKM, where volume change rates dropped to approximately +2∼5% and mass change rates to +1∼3%, consistent with the commercial rubber experimental results. The NBR volume change rate was maintained at +7%, and the mass change rate at +3%, indicating that it is less affected by variations in water content.

### 3.2. Mass and Volume Changes in Rubber Under Different Temperatures in Methanol

**Figure 6:**
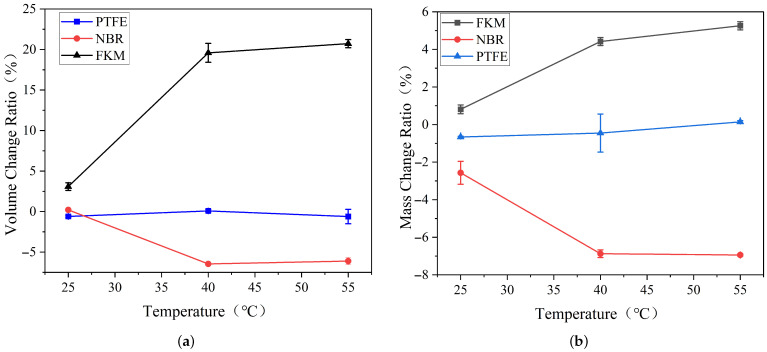
Volume and mass changes in samples under different temperature conditions: (**a**) Volume change rate vs. temperature; (**b**) Mass change rate vs. temperature.

Based on the operating conditions of refined oil pipelines [26], the experimental parameters were controlled with a water content of 5% in the methanol solution, a pressure of 4 MPa, and a pH value of 7. The temperature of the methanol solution was set to 25 °C, 40 °C, and 55 °C. The volume and mass changes in the samples under these different temperature conditions were obtained, and the results are shown in Figure 6.

Through swelling tests at 25 °C, 40 °C, and 55 °C, it was found that temperature variations have a significant impact on Fluorocarbon Rubber (FKM) and Nitrile Butadiene Rubber (NBR), whereas Polytetrafluoroethylene (PTFE) showed negligible changes.

Through swelling tests at 25 °C, 40 °C, and 55 °C, For FKM, increasing temperature significantly enhanced swelling in methanol solution. The average volume change increased from 3.08±0.47% at 25 °C to 19.59±1.16% at 40 °C and 20.72±0.50% at 55 °C. The corresponding mass change increased from 0.81±0.23% to 4.42±0.21% and 5.26±0.22%, respectively. These results indicate that elevated temperature promotes methanol absorption and swelling of FKM.

For NBR, the average volume change was 0.20±0.13% at 25 °C, but decreased to −6.46±0.06% and −6.11±0.45% at 40 °C and 55 °C, respectively. The corresponding mass changes were −2.57±0.61%, −6.87±0.21%, and −6.94±0.06%. This indicates that higher temperature promoted mass loss and dimensional shrinkage of NBR, which may be attributed to the accelerated extraction of low-molecular-weight components or additives from the rubber matrix.

In contrast, PTFE exhibited relatively small changes in both volume and mass under the tested temperature conditions. The average volume changes were −0.60±0.12%, 0.08±0.14%, and −0.61±0.90% at 25 °C, 40 °C, and 55 °C, respectively. The corresponding mass changes were −0.67±0.04%, −0.46±1.01%, and 0.14±0.06%. Although relatively larger standard deviations were observed for some PTFE results, the overall magnitude of change remained much lower than that of FKM and NBR.

The swelling of crosslinked polymers is the result of a compromise between the osmotic swelling pressure and the elastic restorative force of the macromolecular chain network [27]. Molecular motion associated with chain flexibility allows solvent molecules to penetrate the polymer. As the temperature rises, segmental motion increases, facilitating greater diffusion of solvent molecules, which leads to exacerbated swelling in FKM. In the case of NBR, the rise in temperature accelerates the migration of low-molecular-weight polymers and plasticizers out of the rubber matrix, resulting in a further reduction in its mass and volume.

### 3.3. Thermogravimetric Analysis Results of Rubber After Immersion in Methanol with Different Water Contents

**Figure 7:**
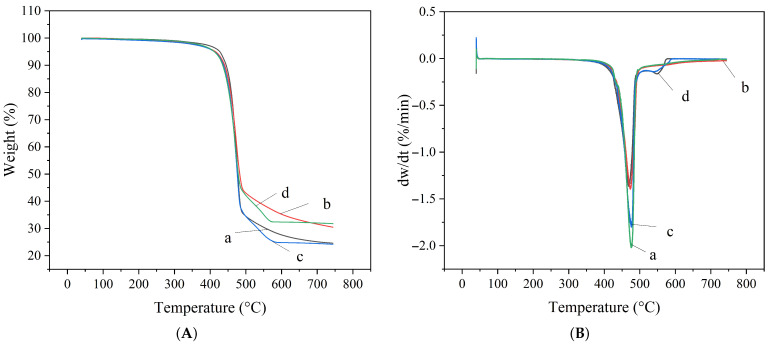
TG-DTG curves of FKM under different water contents (a: 0%, b: 10%, c: 30%, d: 60%): (**A**) TG curves; (**B**) DTG curves.

**Figure 8:**
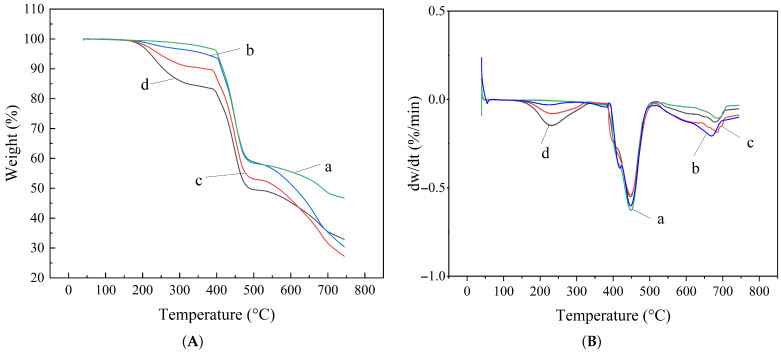
TG-DTG curves of NBR under different water contents (a: 0%, b: 10%, c: 30%, d: 60%): (**A**) TG curves; (**B**) DTG curves.

**Figure 9:**
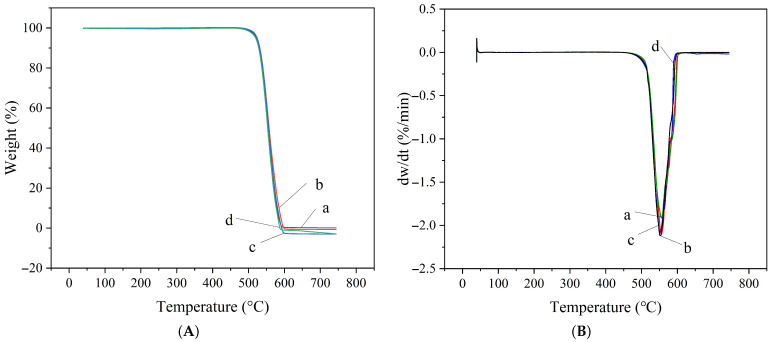
TG-DTG curves of PTFE under different water contents (a: 0%, b: 10%, c: 30%, d: 60%): (**A**) TG curves; (**B**) DTG curves.

Thermogravimetric analysis (TGA) was performed on the three types of sealing rings after being immersed for 7 days at room temperature in methanol solutions with varying water contents. The results are presented in Figure 7, Figure 8 and Figure 9.

As shown in the TG-DTG curves of FKM after immersion in methanol with varying water contents (Figure 7), the thermal decomposition of FKM primarily occurs in the range of 430 °C to 520 °C. Under conditions of 0%, 10%, 30%, and 60% water content, the peak temperatures of weight loss were 476 °C, 474 °C, 476 °C, and 470 °C, respectively. The peak temperatures remained basically stable, indicating that variations in water content have a minimal effect on the thermal decomposition temperature of FKM. This suggests that the swelling of FKM caused by methanol does not significantly alter its chemical structure, nor does it noticeably affect its thermal stability or lead to the destruction of its molecular structure.

The analysis of the TG-DTG curves for NBR after immersion in methanol with 0%, 10%, 30%, and 60% water content (Figure 8) reveals that its thermal decomposition process exhibits a three-stage characteristic:

Stage 1 (140–340 °C): This stage corresponds to the volatilization or decomposition of small-molecule additives or residual solvents within the NBR. The decomposition peak for small organic molecules is most significant at 60% water content. As the water content decreases, the intensity of this peak gradually weakens and completely disappears after immersion in pure methanol. This indicates that pure methanol has a strong capability to extract small organic molecules from the rubber matrix.

Stage 2 (370–540 °C): This is the characteristic decomposition stage of the NBR main chain, corresponding to the thermal degradation of butadiene and acrylonitrile segments. The peak temperatures of weight loss under different water content conditions remained stable within the range of 447 °C to 449 °C, with fluctuations not exceeding 2 °C.

Stage 3 (540–720 °C): This stage likely involves the further oxidative decomposition of residual carbonaceous materials. These findings demonstrate that methanol immersion induces only physical swelling or extraction in NBR without destroying its polymer backbone.

The TG-DTG curves of PTFE after immersion in methanol with 0%, 10%, 30%, and 60% water content (Figure 9) show a weight loss range of 500 °C to 600 °C with a single weight loss peak. The peak temperatures for the different water contents were 551 °C, 555 °C, 555 °C, and 549 °C, respectively. The peak position remained essentially unchanged, confirming that methanol has almost no effect on the chemical structure of PTFE.

### 3.4. FTIR Spectroscopy Results of Rubber After Immersion in Pure Methanol

**Figure 10:**
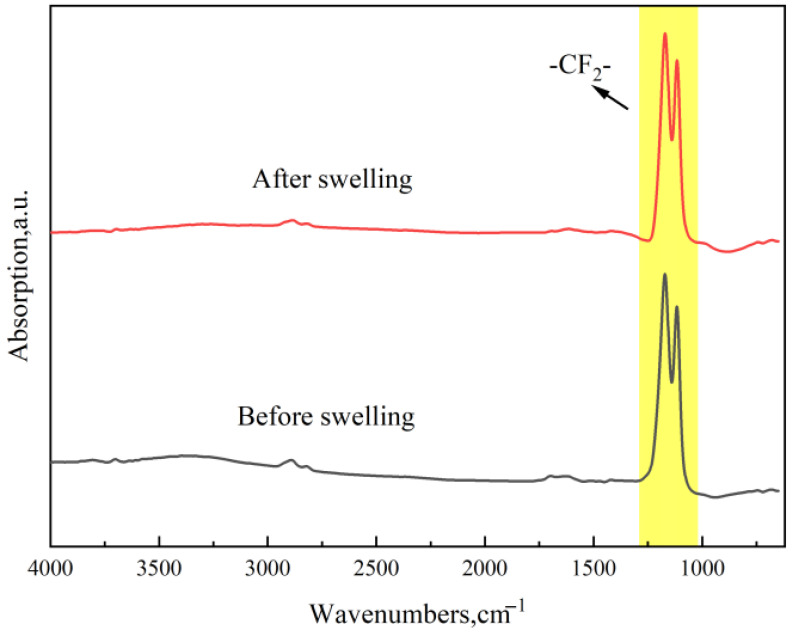
FTIR spectra of PTFE before and after immersion.

**Figure 11:**
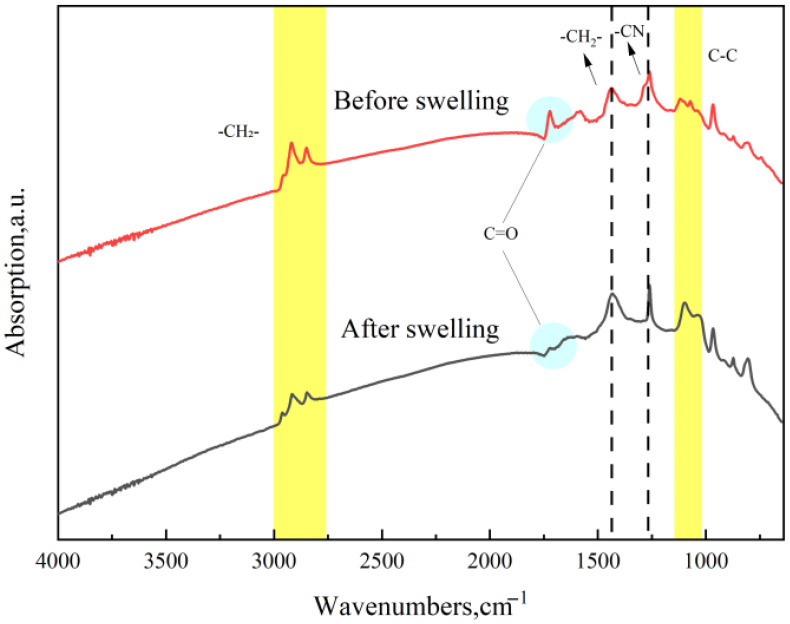
FTIR spectra of NBR before and after immersion.

**Figure 12:**
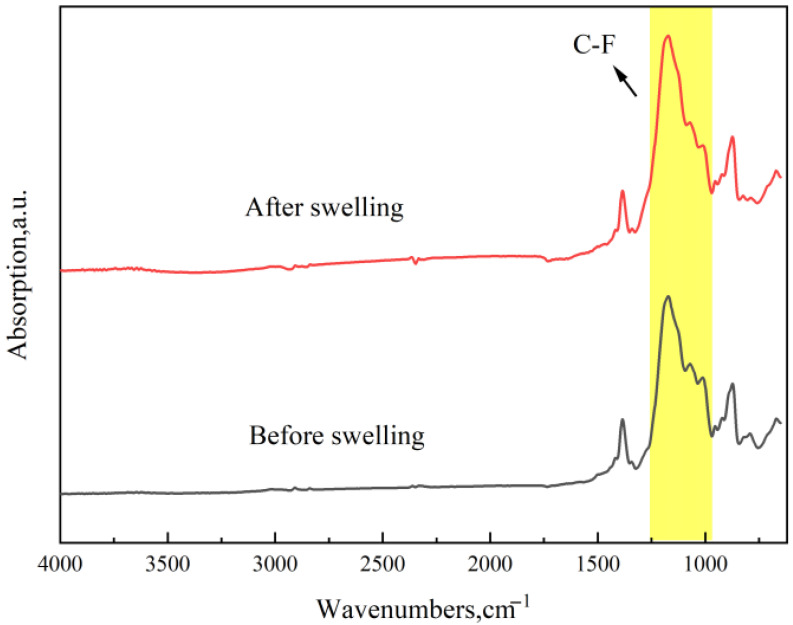
FTIR spectra of FKM before and after immersion.

The three types of sealing rings were immersed in pure methanol at 55 °C for 7 days, after which they were subjected to FTIR analysis. The results are presented in Figure 10, Figure 11 and Figure 12.

As shown in Figure 10, strong characteristic peaks are observed around 1200 cm^−1^ and 1140 cm^−1^. The peak positions remained essentially unchanged before and after immersion, indicating that the skeletal structure of PTFE remained stable in both states. No new functional groups were generated, nor did bond scission or rearrangement occur. The slight variations in absorbance intensity of the major peaks in the post-immersion curve may be attributed to changes in the sample’s surface state (such as surface adsorption or pore structure variations), which could affect the optical path or scattering properties.

The FTIR spectra of NBR before and after methanol immersion are shown in Figure 11. The peaks near 3000 cm^−1^ correspond to -CH_2_- characteristic peaks. In the 1500–1000 cm^−1^ region, the absorbance and shape of characteristic peaks such as -CH_2_- (1437.1 cm^−1^), C-N (1281.9 cm^−1^), C-C (1094.5 cm^−1^), and C=C (870.8, 803.5 cm^−1^) showed no significant changes, suggesting that the macromolecular structure of NBR remained intact after methanol immersion.

However, it is worth noting that the peak intensity at 1700 cm^−1^, typically associated with the vibration of carbonyl groups (C=O), decreased significantly. This indicates that small-molecule compounds containing carbonyl groups, such as the plasticizer Bis(2-ethylhexyl) phthalate, were likely extracted from the NBR matrix by methanol immersion.

The FTIR spectra of FKM before and after immersion are shown in Figure 12. The absorption peaks at 1171.33 cm^−1^ and 873.15 cm^−1^ are associated with C-F vibrations, while the peak near 1385.34 cm^−1^ corresponds to the symmetric deformation vibration of -CH_3_. None of these peaks exhibited significant changes, demonstrating that methanol swelling does not alter the molecular structure of FKM. This finding is consistent with the conclusions drawn in the subsequent GC-MS analysis.

### 3.5. GC-MS Test Results

To verify the substances leached from the rubber materials upon methanol immersion, GC-MS analysis was conducted on the methanol solutions corresponding to the three rubber types after 7 days of immersion at 50 °C. The testing results are presented below.

**Figure 13:**
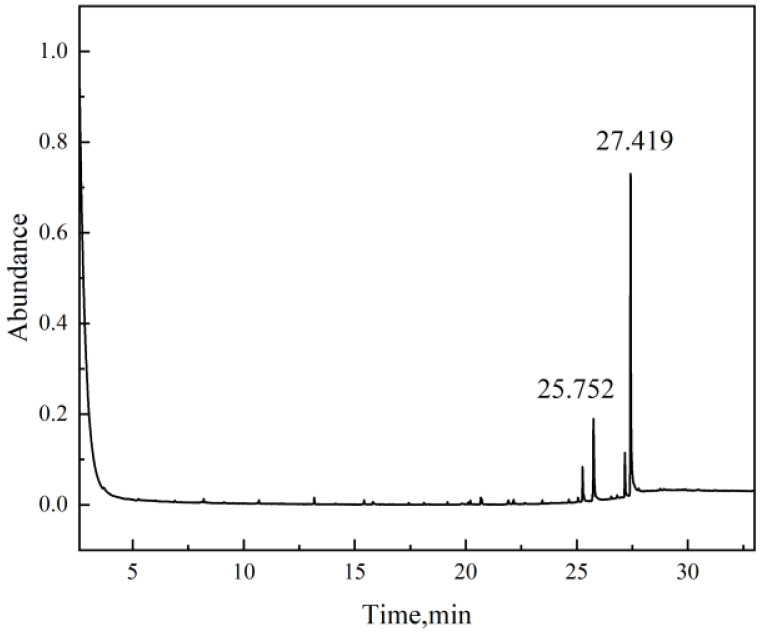
Chromatogram of FKM immersion fluid.

In the case of FKM (Figure 13 and Table 3), the detected compounds were primarily Triphenylphosphine Oxide and Erucamide, which are identified as residual processing aids and demolding agents rather than degradation products of the polymer backbone. Notably, no fluorine-containing fragments were detected in the solution, providing compelling evidence that the chemical integrity of the FKM main chain remains intact. This corroborates the FTIR and TGA results, confirming that the severe swelling of FKM in pure methanol is a purely physical process driven by thermodynamic compatibility (solubility parameter matching), without irreversible chemical corrosion.

**Figure 14:**
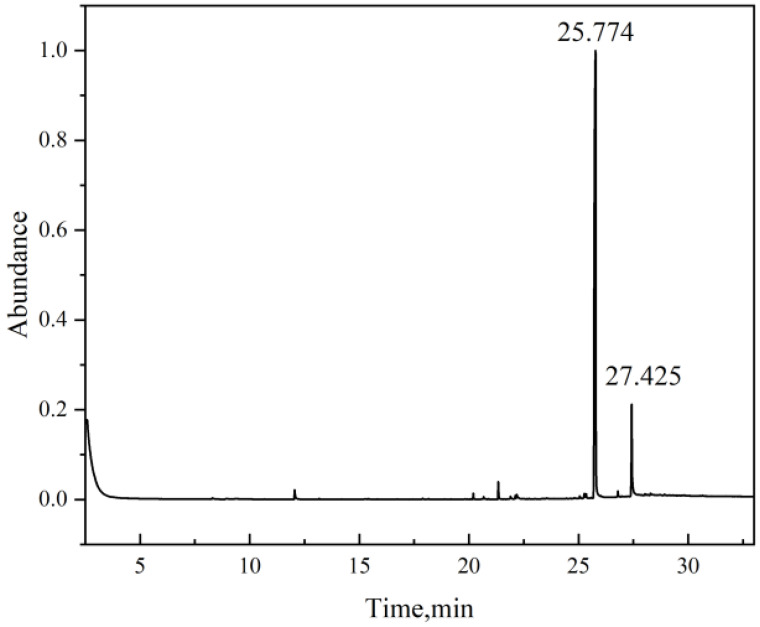
Chromatogram of NBR immersion fluid.

The GC-MS analysis of the NBR immersion fluid (Figure 14 and Table 4) revealed Bis(2-ethylhexyl) phthalate as the predominant leachate with a relative abundance of 100%. As a critical plasticizer used to increase the free volume and flexibility of the NBR network, its extraction by methanol provides the molecular mechanism for the observed macroscopic shrinkage. The high polarity of methanol facilitates the solvation and subsequent migration of the polar phthalate molecules out of the rubber matrix. Critically, this ‘extraction mechanism’ implies not only dimensional contraction but also a significant risk of material embrittlement. The loss of plasticizers typically leads to an increase in glass transition temperature ($T_{g}$) and a reduction in elasticity, rendering the sealing ring susceptible to brittle fracture under the high-pressure fluctuations of long-distance pipelines.

**Figure 15:**
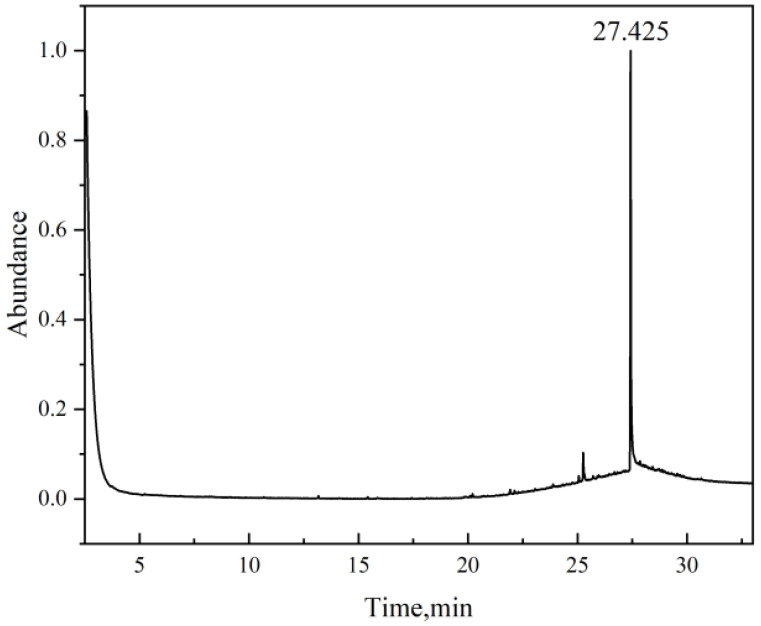
Chromatogram of PTFE immersion fluid.

The chromatogram for PTFE (Figure 15 and Table 5) was remarkably clean, identifying only trace amounts of the surface lubricant Erucamide. The absence of any polymer-derived leachates underscores the exceptional chemical inertness of the C-F bond structure and high crystallinity of PTFE. This ‘zero-extraction’ characteristic makes PTFE the ideal benchmark material for pure methanol environments, ensuring that the conveyed medium remains free from contamination.

## 4. Conclusions

Research into the swelling behavior of sealing materials is of paramount importance for the field of long-distance methanol pipelines. Within the investigated water-content range of 0–20% and temperature range of 25–55 °C, the swelling behavior of FKM, NBR, and PTFE in methanol and methanol–water mixtures was systematically evaluated. The key findings are as follows:

1. Fluorocarbon Rubber (FKM): FKM showed severe swelling in pure methanol, with the volume change ratio reaching approximately 80%, far exceeding the acceptable industrial standard. Based on Hansen solubility parameter analysis, methanol and FKM exhibit poor thermodynamic compatibility; however, methanol can still diffuse into the FKM network because of its small molecular size and high diffusivity, causing plasticization and significant swelling. As the water content increases, the Hansen distance between the methanol–water mixed solvent and FKM increases, reducing solvent–polymer affinity and suppressing methanol uptake. When the water content reaches 5%, the volume change ratio decreases from approximately 80% to about 3%, and the mass change ratio decreases from approximately 30% to about 1%. Elevated temperature may further enhance solvent diffusion and molecular chain mobility, thereby aggravating FKM swelling.
2. Nitrile Butadiene Rubber (NBR): Upon immersion in pure methanol, NBR exhibits a decrease in both volume and mass. This is due to the strong polarity of methanol, which forms hydrogen bonds with the polar groups in NBR, causing the extraction of low-molecular-weight polymers and plasticizers. However, with increasing water content, water molecules form a hydrogen bond network with methanol, reducing the overall polarity of the solution and allowing the mass and volume of NBR to recover. Elevated temperatures accelerate the migration of substances within NBR, leading to further reductions in mass and volume.
3. Polytetrafluoroethylene (PTFE): Due to its highly symmetrical and rigid molecular chains, high crystallinity, and non-polar nature, PTFE shows almost no swelling in methanol solutions, demonstrating excellent chemical stability.
4. Microscopic Mechanism: Results from TGA, FTIR, and GC-MS indicate that the interaction between methanol and the three rubber materials is primarily characterized by physical swelling or extraction, without destroying the polymer backbone structure.
  - FKM: After immersion in methanol with varying water contents, the thermal decomposition temperature remained stable, and the characteristic C-F peaks in the FTIR spectra showed no shifts. Only additives such as Triphenylphosphine Oxide and Erucamide were detected as leachates, confirming that the chemical structure remained intact.
  - NBR: The TGA stage corresponding to small-molecule volatilization (140–340 °C) diminished as water content decreased (i.e., higher methanol concentration), indicating that methanol effectively extracts these compounds. While the characteristic FTIR peaks for -CH_2_- and -CN remained unchanged, GC-MS detected the leaching of small molecules, specifically the plasticizer Bis(2-ethylhexyl) phthalate, confirming the extraction mechanism.
  - PTFE: The single weight loss peak at 500–600 °C and the characteristic C-F peaks (1200 cm^−1^, 1140 cm^−1^) remained unchanged after immersion. Only the processing aid Erucamide was detected in the leachate, further verifying its structural stability.
5. Future Work: Future work may further integrate experimental swelling data with data-driven modeling to predict the behavior of sealing materials under varying methanol composition, water content, temperature, pressure, and exposure time. Machine-learning and neural-network-based methods could help optimize material selection and reduce trial-and-error testing in methanol pipeline applications. In addition, advanced self-healing elastomers and polymer nanocomposites may provide promising strategies for improving long-term sealing reliability. Intrinsic self-healing mechanisms based on reversible dynamic bonds or supramolecular interactions, as well as extrinsic systems based on microcapsule-type healing agents, could potentially compensate for swelling-induced damage, cracking, plasticizer loss, and embrittlement. Future studies should also consider cyclic swelling/deswelling, fatigue behavior, long-term degradation, dynamic flow, pressure fluctuations, and multi-component methanol fluids to better simulate real pipeline conditions.

## Figures and Tables

**Table 1:** Hansen solubility parameters of methanol, water, and FKM-26.

Material	$\delta_{D}$	$\delta_{P}$	$\delta_{H}$	$\delta_{t}$
	(MPa^1/2^)	(MPa^1/2^)	(MPa^1/2^)	(MPa^1/2^)
Methanol	15.1	12.3	22.3	29.61
Water	15.5	16.0	42.3	47.81
FKM-26 (approx.)	14.6	10.0	1.6	17.77
$R_{o}$ (FKM-26, approx.)	–	–	–	8.8

**Table 2:** Calculated Hansen distance ($R_{a}$) and RED values for methanol–water systems.

Solvent System	Water Fraction (*x*)	$R_{a}$ (MPa^1/2^)	RED
Methanol	0.0	20.85	2.37
Methanol/water	0.1	22.88	2.60
Methanol/water	0.5	31.01	3.52

**Table 3:** Leachates in FKM immersion fluid.

Name	Time (min)	Formula	CAS	Confidence	Abundance %
Triphenylphosphine Oxide	25.752	C_18_H_15_OP	791-28-6	93.69	31.56
Erucamide	27.419	C_22_H_43_NO	112-84-5	95.09	100

**Table 4:** Leachates in NBR immersion fluid.

Name	Time (min)	Formula	CAS	Confidence	Abundance %
Bis(2-ethylhexyl) phthalate	25.774	C_24_H_38_O_4_	117-81-7	94.72	100
Erucamide	27.425	C_22_H_43_NO	112-84-5	94.36	10.36

**Table 5:** Leachates in PTFE immersion fluid.

Name	Time (min)	Formula	CAS	Confidence	Abundance %
Erucamide	27.425	C_22_H_43_NO	112-84-5	94.9	100

## Data Availability

The original contributions presented in the study are included in the article, further inquiries can be directed to the corresponding author.
